# Substances with zero static permittivity

**DOI:** 10.1038/s41598-022-11454-8

**Published:** 2022-05-06

**Authors:** Vladimir F. Kharlamov

**Affiliations:** Orel State University, 302026 Orel, Russia

**Keywords:** Materials science, Mathematics and computing, Nanoscience and technology, Physics

## Abstract

The structure of materials with a negative electric susceptibility has been determined. Their electrical properties correspond to vanishing static dielectric permittivity. We give a theoretical explanation of anomalous polarization of powders observed in experiments and the mechanism of emergence in an aerosol cloud of an electric field, which is responsible for spark discharges (lightnings). We also explain the ball lightning properties known from observations. A fundamental scientific problem has been solved and the grounds have been laid for applications of new principles in electronics and electrical engineering.

## Introduction

When a medium is polarized by an electric field, the external charge or the field induction δ**D** determine the external action, and the result of this action is determined by the field strength δ**E** in the medium. The dielectric permittivity ε(ω, **k**) of the medium relates δ**D** and δ**E**:

$$\updelta{\bf E} \left( {\upomega ,{\mathbf{k}}} \right) \, = \updelta {\mathbf{D}}\left( {\upomega ,{\mathbf{k}}} \right)/\upvarepsilon \left( {\upomega ,{\mathbf{k}}} \right)$$

The quantity 1/ε(ω, **k**) satisfies the Kramers–Kronig dispersion relation, which accounts for the loss of electron energy *W* and the change in momentum **k** during their scattering in the medium. The static permittivity satisfies the equality 1/ε = 1 − *I*, where ε = ε(0, **k**);

$$I = 2\int\limits_{0}^{\infty } {\sigma_{{\text{k}}} } (W,{\mathbf{k}})\frac{dW}{W} \ge 0$$

σ_k_(*W*, **k**) is the quantity associated with the form factor of inelastic electron scattering. It follows from *I* ≥ 0 that the static permittivity of any substance satisfies one of the following conditions: ε ≥ 1 or ε ≤ 0^1,2^. The inequality *I* < 0 cannot hold due to the electron scattering, and, therefore, the values 0 < ε < 1 are not possible.

The existence of substances that satisfy the condition ε ≤ 0 is a debatable subject^1,2^. There are no references in the literature to materials with a negative electric susceptibility χ = ε − 1.

The purpose of the present work is to provide a theoretical description of the structure and electrical properties of materials with a negative electric susceptibility.

## Results

Consider an aerosol, consisting of suspended in air spherical micro particles of an isotropic dielectric, placed in a uniform electric field **E**_0_. We assume that ionized donor centers are uniformly distributed over the particle surface and the bulk volume contains free electrons. Let us determine the change in the aerosol free energy due to the polarization by an electric field.

According to Gauss’s theorem, the strength of the field, created in the bulk of a micro particle by the positive surface charge of ionized donor centers, is zero. The electric field potentials of any two points inside the particle volume are equal. Therefore, the displacement of a free electron inside the volume does not change the particle energy. There are no forces inside the particle bulk volume that counteract the electron displacement. Therefore, under the action of an external field, the free electrons are displaced to the maximum distance to reside in the near-surface region. The work of the field forces is relatively small. It provides the displacement of electrons in the bulk and creation of the particle local field by the polarized charges.

Let us calculate the field-induced dipole moment *p* of a spherical particle, assuming that it is placed in a uniform electric field of the strength **E**_0_. Inside the particle volume, we use the Maxwell equation div**D**_b_ = *ne*, where **D**_b_ is the field electric induction, *n* is the concentration of free electrons, and *e* is the electron charge. The electrons are in the state of equilibrium, therefore, the following condition is met: **E**_0_ + **E**_b_ = 0, where **E**_0_ is a constant vector; **E**_b_ is the field strength created by polarizing charges. It follows from here that **E**_b_ is a constant vector in the particle bulk volume, and div**D**_b_ = div(ε_b_ε_0_**E**_b_) = 0, *n* = 0. It means that there are no free electrons in the bulk volume of a polarized spherical particle since they are all distributed over its surface. From the condition *E*_b_ = *C*, where *C* is a constant, it follows that the bulk electric field is uniform:

$$E_{{\text{b}}} = \frac{\partial \upvarphi }{{\partial z}} = C;\quad \upvarphi = Cz$$

where φ is the bulk potential; the *z-*axis is directed along the dipole axis. The field potential φ = *Cz* satisfies the Laplace equation with the boundary condition φ = φ_s_. Assuming *C* = *P*_b_/(3ε_0_), where *P*_b_ = *p*/*v* is the ball polarization; *v* is the ball volume, one can determine the field potential on the ball surface:

1 $$\upvarphi_{{\text{s}}} = Cz = \frac{{P_{{\text{b}}} z}}{{3\upvarepsilon_{0} }} = \frac{{vP_{{\text{b}}} z}}{{4\pi \upvarepsilon_{0} r^{3} }} = \frac{p\cos \uptheta }{{4\pi \upvarepsilon_{0} r^{2} }}$$

where *r* is the ball radius; θ is the angle between the dipole axis and the radius vector drawn from the ball center to a point on the surface. In accord with the uniqueness theorem, we have obtained a general solution to the problem of the distribution of potential inside the volume and on the surface of a polarized spherical particle.

Condition (1) corresponds to the following distribution of the density of the surface charge created by free electrons and ionized donor centers:

$$\upsigma (\theta ) = \upsigma_{m} \cos \uptheta = P_{b} \cos \uptheta$$

The maximum surface density of the positive charge of ionized donor centers σ_m_ = *en*_s_, where *n*_s_ is the concentration of ionized donor centers on the particle surface. The same value has the maximum surface density of the particle negative charge. So, the particle polarization density is *P*_b_ = σ_m_ = *en*_s_. The dipole moment of the polarized particle does not depend on the field strength:

$$p = vP_{b} = v\upsigma _{m} = ven_{s}$$

Under the action of an electric field of strength **E**_0_, free electrons of the cloud particles experience the maximal displacement. The polarization density of a homogenous disperse system is

2 $${\mathbf{P}} = N\left( {{\mathbf{p}}_{{\text{d}}} + {\mathbf{p}}} \right) = N(\alpha \upvarepsilon_{0} {\mathbf{E}} + p{\mathbf{E}}_{0} /E_{0} ),$$

where *N* is the number of micro particles per unit volume; **p**_d_ is the dipole moment of a dielectric particle brought about by the polarization of atoms due to the displacement of their bound electrons; α is the polarizability of a dielectric particle, α < 3*v*^3^; **E** is the strength of the mean macroscopic field in the bulk of the disperse system; *p* = *ven*_s_. The polarized cloud creates an electric field with the strength.

$${\mathbf{E}} = - \frac{{\mathbf{P}}}{{\upvarepsilon_{0} }} = - \frac{{Np{\mathbf{E}}_{0} }}{{\upvarepsilon_{0} E_{0} (1 + \alpha N)}}$$

Therefore, the following conditions are satisfied:

3 $$\upvarepsilon_{0}{\bf E} + {\mathbf{P}} = 0$$

4 $${\mathbf{D}} = \upvarepsilon_{0} \upvarepsilon{\bf E} = 0$$

where **P** = ε_0_χ**E**; χ is the system’s electric susceptibility; ε is the aerosol dielectric permittivity. The quantities *E* and *P* have self-consistent values. According to (3) and (4), the following equalities hold: χ = –1; ε = 1 + χ = 0.

The change in energy of an aerosol polarized by an electric field in an isothermal process is (see (3)):

$$F_{{\text{e}}} = \left( {{\mathbf{E}},{\mathbf{P}}} \right)/{2} = {-}\upvarepsilon_{0}E^{{2}} /{2}$$

The change in free energy *F* of the cloud due to polarization is equal to the algebraic sum of the electric field energy ε_0_*E*^2^/2 and energy *F*_e_ = –ε_0_*E*^2^/2:

5 $$F = \upvarepsilon \upvarepsilon_{0} E^{{2}} /{2} = F_{{\text{e}}} + \, \upvarepsilon_{0} E^{{2}} /{2} = {-}\upvarepsilon_{0} E^{{2}} /{2} + \upvarepsilon_{0} E^{{2}} /{2} = 0$$

The equality *F* = 0 corresponds to the condition ε = 0. An increase in the field energy ε_0_*E*^2^/2 in (5) is due to a decrease in the energy of the cloud *F*_e_ = –ε_0_*E*^2^/2.

Let us estimate the field strength in the cloud, using (2) and (3):

6 $$-E = P/\upvarepsilon_{0} \cong Np/\upvarepsilon_{0} \cong Nven_{s} /\upvarepsilon_{0} \cong l^{3} en_{s} /\left( {2R^{3} \upvarepsilon_{0} } \right),$$

where *N* = *R*^*–*3^, *R* is the average distance between aerosol particles,* l* is the particle diameter. For example, for *R*/*l* = 10, *n*_s_ = 10^18^ m^–2^, we have *E* = 10^7^ V/m. If *l* = 10 µm, the average value of the concentration of free electrons in a non-polarized spherical particle is *n*_0_ = 6 *n*_*s*_/*l* = 6·10^23^ m^–3^. The field strength *E* = 10^7^ V/m exceeds the critical value at which a spark discharge (lightening) in the cloud occurs. The main property of the polarized aerosol cloud is expressed by the inequality *E* >> *E*_0,_ where *E* is the field strength in the cloud; *E*_0_ is the strength of the external field that causes the cloud polarization.

## Discussion

The obtained theoretical results enable one to explain the phenomena of atmospheric electricity.

Consider the polarization of a small aerosol cloud with a volume of less than 1 m^3^. The polarized cloud is in a metastable state. This polarized state exists independently on the action of the external field. At field strength in the cloud *E* > 10^6^ V/m, the field ignites a corona discharge accompanied by gas glow. If the cloud is in an external field **E**_0_, then the discharge intensity in air depends on the total field strength:

7 $$\textbf{E} _{{\text{s}}} = {\bf E}_{0} +{\bf E}={\bf E} _{0} {-}{\mathbf{P}}/\upvarepsilon_{0}$$

Approaching the cloud to a conductor brings about the appearance of an electric current in it caused by the electric field of the cloud. Due to a decrease in the field strength *E*, the metastable polarization state of the cloud is destroyed.

Based on the observational results over ball lightnings (BL) (see, for example^4,5^), we assume that BL is a polarized aerosol cloud consisting of water micro particles suspended in air. The cloud is in a metastable state. The cloud electric field is an analog of the field of a dielectric ball polarized by a uniform field. This field is uniform inside the ball and coincides with the dipole field outside the ball^3^. The BL glow spectrum contains nitrogen and oxygen lines^4^, which corresponds to the excitation of a gas discharge in air by the BL field. When ball lightning enters the region of the external electric field, because condition (7) holds true, the electric field strength that ignites the discharge changes and it affects the intensity of the BL glow. This is related with a periodic change in the intensity of the BL glow (spectral lines of nitrogen and oxygen) at a 100 Hz frequency of a high-voltage transmission line near which the ball lightning was observed^4^.

Water micro droplets can stay suspended in the air for a long time, forming a fog. The water surface has a negative electrical potential due to the accumulation of hydroxyl ions HO^−^^6^. Hence, the surface of water droplets is negatively charged, while positively charged hydronium ions H_3_O^+^ reside in the bulk volume. Droplet negative and positive ion charges are separated. The applied electric field displaces the droplet bulk H_3_O^+^ ions. The droplet acquires a dipole moment, whose value is determined by the expression *p* = *ven*_si_, where *n*_si_ is the surface concentration of HO^−^ ions. This is the reason of the formation of ball lightning and the occurrence of linear lightning in thunderclouds.

The surface of the Earth is negatively charged, and in clear cloudless weather it creates in the troposphere an electric field of 10^2^ V/m. The Earth’s field in clouds is a hundred times greater. The field of the polarized cloud is similar to the field of a dipole^7^. In thunderclouds, an electric field of the order of 10^7^ V/m is created and spark discharges (lightning) are observed^7–9^. These phenomena correspond to the effect that the concentration *N* of water aerosol micro particles has on the field strength in a cloud (expression (6)). In a cloud polarized by an electric field, the oppositely charged particles, created by cosmic rays and other factors, are separated. It gives rise to the formation of the bulk positive and negative charges that create an electric field^7–9^. This field can be a cause of the cloud polarization accompanied by lightning.

Let us assume in (6) *R*/*l* = 1, which corresponds to the location of spherical micro particles in a powder. We will use expressions (2)–(6) to explain the effects that arise due to the powder polarization.

In the case a sinusoidal field is applied to polarize finely dispersed dielectrics KMnO_4_ and KNaC_4_H_4_O_6_·4H_2_O placed respectively in molecular hydrogen or vacuum, the following phenomenon is observed. After the creation of donor centers on the surface of powder micro particles and the appearance of bulk free electrons, the effect of field amplification and inversion occurs. In this case, the amplitude *J*_m_ current in the electrical circuit increases by 10^4^ times independently of its frequency (0.02–1 kHz). The amplitude *U*_cm_ of the alternating voltage in the electrical circuit decreases by 12–14 times, and the current and voltage are phase shifted by 180°. Ohmic losses are zero. The capacitance of the powder layer has a negative value (*Z* = –*U*_m_/*J*_m_) and does not depend on the frequency of the sinusoidal field. The effect arises under the condition that the powder layer is placed between the dielectric plates and the current through the powder layer is zero^10,11^.

This phenomenon is caused by the phase transition ε_1_ > 1 → ε = 0 in the disperse system and exists under changing external conditions. For example, in the case of polarization of KMnO_4_ by a sinusoidal field in a hydrogen medium, a change in temperature *T* = 295—400 К does not affect the current amplitude *J*_m_ and the value of the capacitance *Z* < 0. With a change in the gas pressure, the values of *J*_m_, *U*_m_, and the dependence *J*(*t*)[*U*(*t*)] change due to the change in the state of the surface of powder micro particles, on which depends the concentration of free electrons (according to measurements conducted by different methods). The limiting maximum value of *J*_m_ at *Z* < 0 corresponds to the linear dependence of *J*(*t*)[*U*(*t*)]^10,11^.

Due to inversion of the field **E = –P**/ε_0_, the electric voltage *U*(*t*) across the polarized powder layer varies in antiphase with the source voltage *U*_c_(*t*). The periodic change of the field energy of the dispersed polarized system occurs in sync with the change of its energy (see (5)). Therefore, the ohmic losses are zero. This effect is due to the exchange–correlation interaction of free electrons^1,2^. The maximum density of polarization charges on the opposite sides of the powder layer σ_pm_ = *P*_m_ is anomalously large. It causes a sharp increase in the current density *j*(*t*) = –∂σ_p_/∂*t* = ε_0_∂*E*/∂*t*, which corresponds to an increase in the field amplitude *E*_m_ in powder by 10^4^ times (|*E*_m_|/*E*_0m_ = 10^4^). This effect does not depend on the frequency of the alternating field and is indicative of the fulfillment for powders of the condition ε = 0.

In a similar way, longitudinal plasma waves arise in semiconductors under the action of an electromagnetic field. It follows from the Maxwell and the continuity equations that longitudinal waves arise when the condition ε_s_(ω_p_) = 0 is met, where ε_s_(ω_p_) is the complex permittivity of the semiconductor; ω_p_ is the electromagnetic field plasma frequency. The real part of the electrical conductivity must be negligibly small, which corresponds to microwave field frequencies. The magnetic field of the electromagnetic wave can be neglected^12^. There are known composites and metamaterials with negative and zero values of ε. They differ from the powders that we have studied by the frequency dispersion ∂ε/∂ω, ohmic losses and the absence of the effect of amplification and inversion of a zero or low-frequency field^13–15^.

There has been experimentally observed spontaneous polarization of finely dispersed dielectrics KMnO_4_, Pb (NO_3_)_2_, and CsNO_3_ after the creation of donor centers by hydrogen atoms on the surface of their micro particles and the appearance of bulk free electrons^16^. Spontaneously polarized powder creates an electric field. In a closed circuit, it serves as a source of electric current^17^. Spontaneous polarization of the powder consists in a spontaneous displacement of free electrons in the particles of the disperse system, so that the dipole moments of the particles are oriented in the same direction. The powder polarization charges create an electric field with strength **E = –P**/ε_0_. This field polarizes atoms of the dielectrics by displacing their bound electrons. Therefore, the condition **P = P**_c_ + **P**_d_ holds true, where **P**_c_ is the spontaneous polarization density of the powder layer due to the displacement of free electrons; **P**_d_ = *N*αε_0_**E** is the polarization density of dielectric particles by the field **E.** We have

8 $${\mathbf{P}}_{{\text{c}}} = {-}\upvarepsilon_{0} {\mathbf{E}}{-}N\alpha\upvarepsilon _{0} {\mathbf{E}}$$

9 $$\upchi _{{\text{c}}} = {-}({1} + N\alpha ),$$

where χ_c_ is the powder electric susceptibility. Using (8), we get

$$F_{ec} = \left( {{\mathbf{E}},{\mathbf{P}}_{c} } \right)/2 = {-}(1 \, + N\alpha )\upvarepsilon_{0}E^{2} /2$$

10 $$F_{{\text{c}}} = \upvarepsilon_{c} \upvarepsilon_{0} E^{{2}} /{2} = F_{{{\text{ec}}}} + \upvarepsilon_{0} E^{{2}} /{2} = {-}N\alpha \upvarepsilon_{0}E^{{2}} /{2}$$

where *F*_ec_ and *F*_c_ are respectively the internal and free energies of the spontaneously polarized powder. Its permittivity is negative: ε_c_ = – *N*α. According to (8) and (10), the macroscopic electric field arises in the powder due to the fulfillment of the process spontaneity condition *F*_c_ < 0, and the positive feedback between *P*_c_ and *E.* The field strength that limits the displacement of free electrons in the powder particles is reduced due to polarization of dielectric atoms (see (8)): *E* = *P*_c_[ε_0_(1 + *N*α)]^–1^. It corresponds to an increase in the absolute value of susceptibility χ_c_ (see (9)).

## Conclusion

A dielectric micro particle with ionized donor centers on its surface and free electrons in the bulk has unique electrical properties. The magnitude of the micro particle field-induced dipole moment is anomalously large and does not depend on the magnitude of the field strength. The polarized disperse system consisting of such particles (powder or aerosol) creates an electric field with the strength **E = −P**/ε_0_. The quantities *E* and *P* have self-consistent values. We give a theoretical explanation of anomalous polarization of disperse systems. A disperse system may exhibit the effects of electric field amplification and inversion, spontaneous polarization, and the formation of a metastable polarization state which can exist independently on the action of an external field. Ball lightning and spontaneously polarized powder are sources of electromotive force of the same nature. In both cases, the electric field is created by polarization charges of the disperse system in a metastable polarization state in which *F*_c_ < 0*.*

Spontaneously polarized powder can find applications in current sources similar to thermoelectric converters.
